# Managing Placenta Accreta Spectrum: Diagnosis, Surgical Strategies, and Postoperative Care

**DOI:** 10.7759/cureus.86271

**Published:** 2025-06-18

**Authors:** Atif Bashir Fazari, Ufwana Javid, Asma Fahad, Shaima AlSuwaidi, Fareeda Nikhat

**Affiliations:** 1 Obstetrics and Gynecology, Latifa Hospital, Dubai Health, Dubai, ARE; 2 Obstetrics and Gynecology, Dubai Health, Dubai, ARE

**Keywords:** antepartum hemorrhage, focal accreta, focal placenta accreta, placenta previa, previous cesarean section

## Abstract

Placenta accreta spectrum (PAS) refers to a group of disorders characterized by abnormal trophoblastic invasion of the uterine wall, which can result in life-threatening complications such as severe hemorrhage and the need for hysterectomy. A 30-year-old female patient with a history of two prior cesarean sections and diagnosed with Grade IV placenta previa at 34+0 weeks gestation was referred to our hospital for management. The patient presented with antepartum hemorrhage and was at elevated risk for PAS due to her previous cesarean sections and placenta previa. Ultrasound findings suggested PAS, including hypervascularity, consistent with trophoblastic invasion. Elective cesarean section was performed, and intraoperatively, focal accreta was identified with manual removal of the placenta and bilateral uterine artery ligation to control hemorrhage. Histopathology confirmed myometrial tissue with hemorrhage and dilated blood vessels, consistent with PAS, but without evidence of malignancy. The patient was managed in a multidisciplinary setting and received postoperative care for bleeding complications related to Factor 12 deficiency. Her recovery was uneventful, and she was discharged on postoperative day 3. This case highlights the importance of early diagnosis, imaging, and multidisciplinary management in optimizing maternal and fetal outcomes in PAS.

## Introduction

Placenta accreta spectrum (PAS) is a general term used to describe abnormal trophoblast invasion into the myometrium, and sometimes to or beyond the serosa (can be classified into placenta accreta, increta, and percreta) [1]. It is clinically important because the placenta does not spontaneously separate at delivery, and attempts at manual removal result in hemorrhage, which can be life-threatening and usually necessitates hysterectomy in up to 52% of cases [2]. The pathogenesis of most cases of PAS is thought to be placental implantation at an area of defective decidualization caused by preexisting damage to the endometrial-myometrial interface [3,4].

Placenta previa and previous cesarean sections are well-established risk factors for PAS, as the scar tissue from prior cesareans can disrupt the normal decidualization of the endometrium, making it more likely for the placenta to abnormally invade the myometrium [3].

The global incidence of PAS disorders is on the rise, largely attributed to the growing number of cesarean deliveries performed worldwide (1 in 5 of all deliveries worldwide). From 1 in 20,000 births in 1951, the incidence reached 1 in 2500 and 1 in 533 between 1982 and 2002, respectively [2]. This number is set to continue increasing over the coming decade, with nearly a third (29%) of all births likely to take place by caesarean section by 2030, the research finds [5].

This case involves a 30-year-old female patient with a history of two previous cesarean sections who was diagnosed with placenta previa Grade IV at 34+0 weeks gestation. The patient’s history, including prior cesarean deliveries, placed her at higher risk for complications such as PAS, a condition characterized by abnormal trophoblast invasion into the myometrium, which can result in significant hemorrhage and complications during delivery [5]. The diagnosis of PAS was suspected in this case based on imaging findings, clinical risk factors, and intraoperative findings [6].

We present the intraoperative diagnosis and management of this case which shows focal accreta with invasion of the myometrium by placental sinuses in a localized area of the anterior uterine wall.

## Case presentation

We present the case of a 30-year-old woman with a history of two term cesarean sections diagnosed with Grade IV placenta previa, a condition in which the placenta completely covers the cervical os. The patient also had Factor 12 deficiency, which further complicated her case due to an increased risk of bleeding.

The patient was booked in our facility (Latifa Hospital, UAE) and planned for elective cesarean section at 35 weeks gestation (late booking and presentation).

At 34+4 weeks gestation, the patient presented with antepartum hemorrhage (APH), a common complication in cases of placenta previa and a concerning symptom that often necessitates prompt management. She was admitted under observation and multidisciplinary management, which included consultation with obstetric medicine, hematology, anesthesia, and the fetal medicine unit.

**Figure 1:**
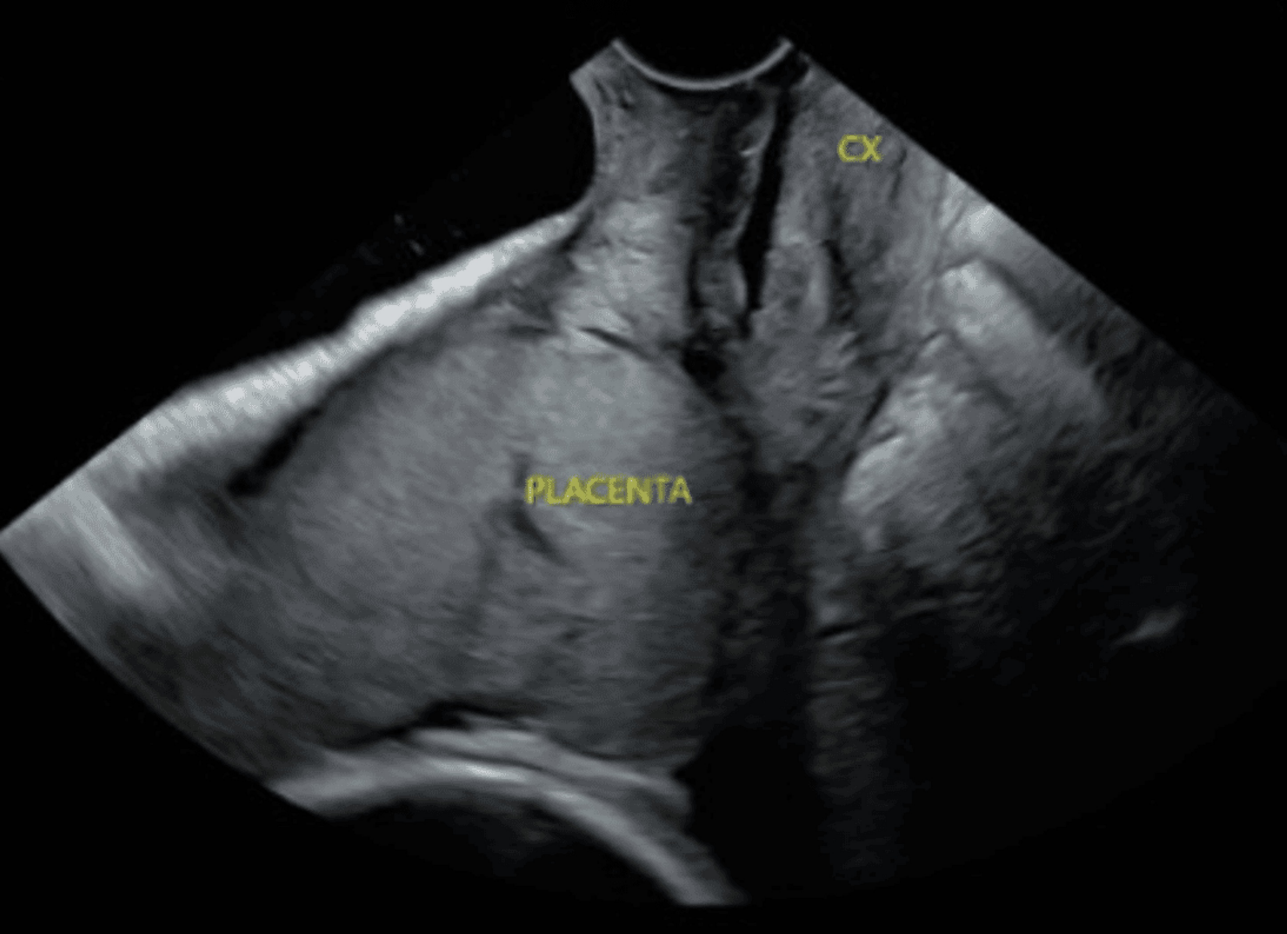
Ultrasound image showing the placenta completely covering the internal cervical os

A detailed ultrasound (US) scan was done in the maternal fetal medicine unit; findings were significant for an anterior placenta completely covering the cervical os (see Figure 1).

**Figure 2:**
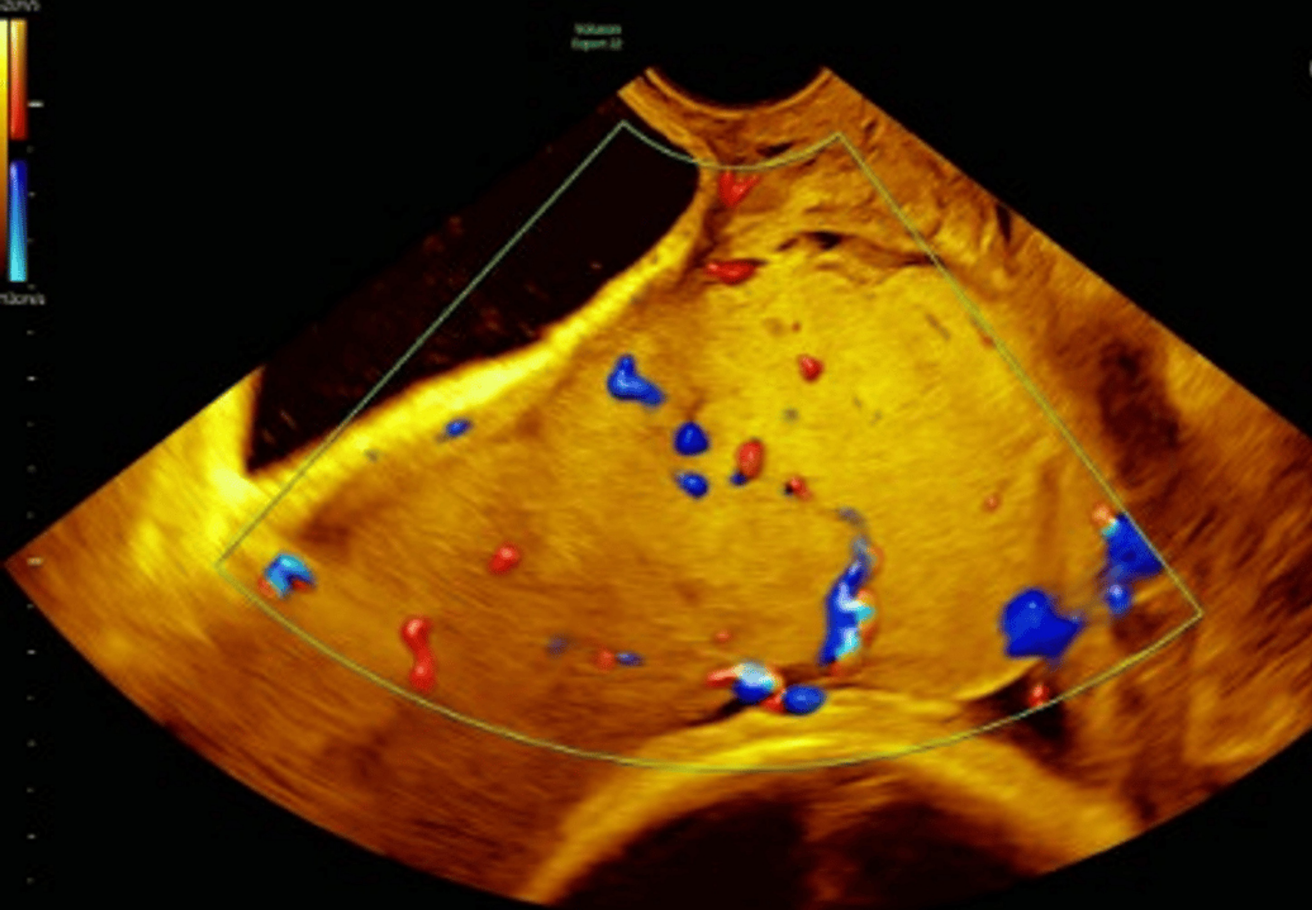
Ultrasound image showing a smooth placental surface with occasional lacunae and hypervascularity noted outside the placenta, particularly on the right side of the lower uterine segment and cervix

Additional findings included a well maintained retroplacental hypoechogenic line, a smooth placental surface with occasional lacunae, and hypervascularity noted outside the placenta, particularly on the right side of the lower uterine segment and cervix (see Figure 2).

The absence of bridging vessels on US, however, was reassuring as it suggested that there was no evidence of a complete invasion through the serosa or extension beyond the myometrium.

In view of APH and the US findings, the patient was prepared for cesarean section after being counseled about all the risks associated with her surgery.

The surgery was performed with the recommended care bundle of high-risk PAS cases with senior obstetrician and anesthetists present in the OT and a vascular surgeon on standby. Blood and blood products were on standby.

**Figure 3:**
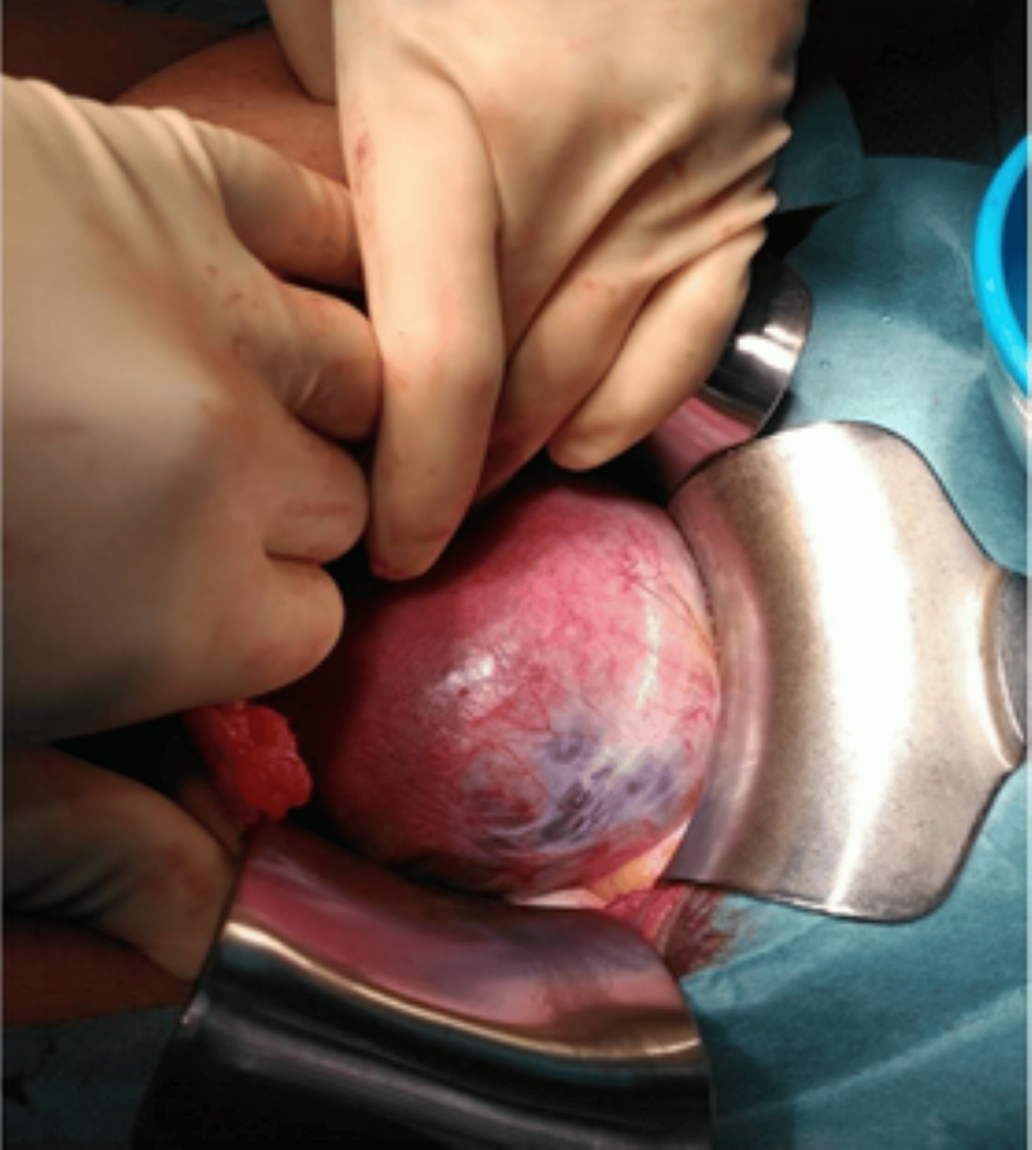
An area of focal adherence of the placenta, identified in the anterior uterine wall on the right side

Intraoperatively, a Pfannenstiel incision was made and the abdominal wall was opened in layers. An area of focal adherence of the placenta was identified in the anterior uterine wall on the right side (see Figure 3).

**Figure 4:**
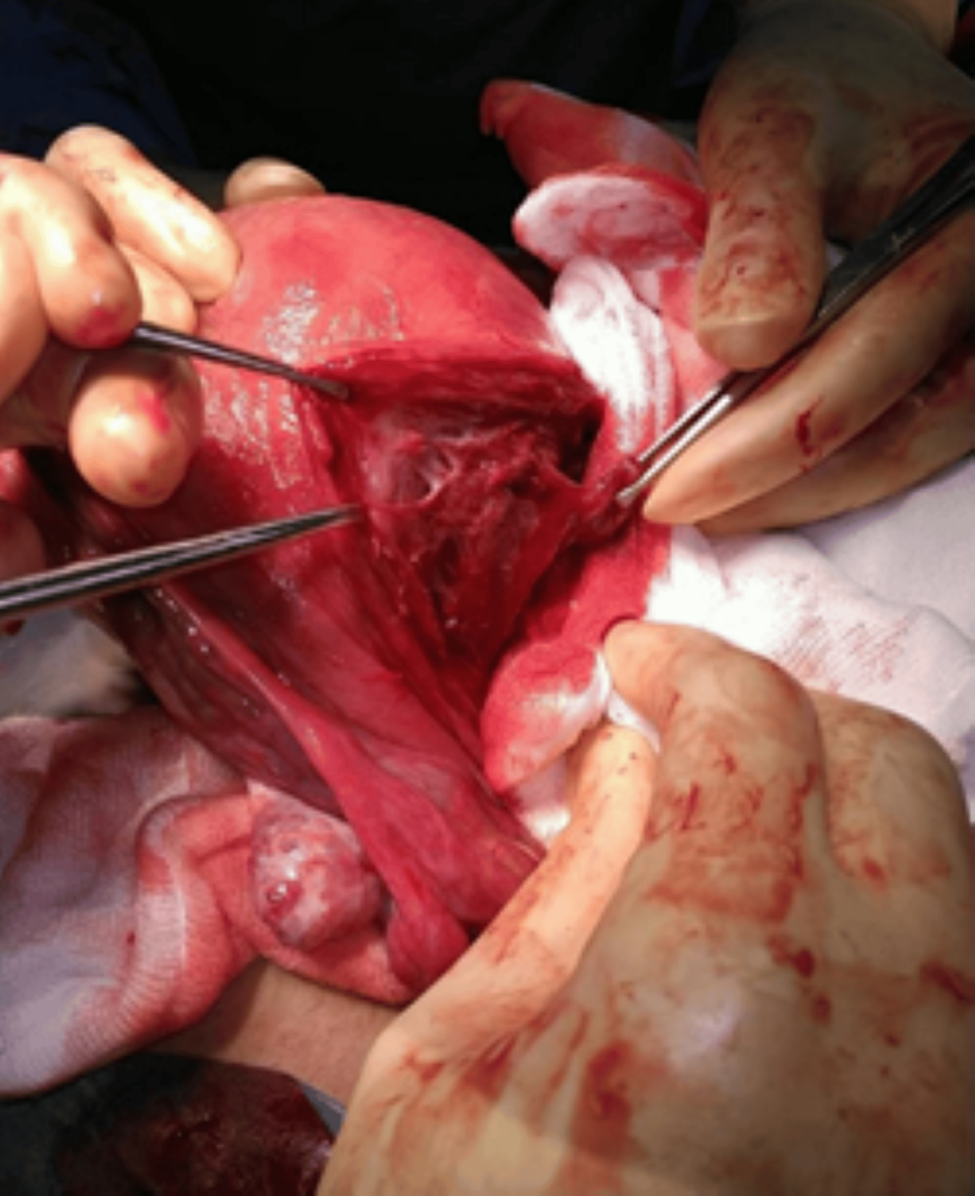
Placenta sinuses in the uterine myometrium

**Figure 5:**
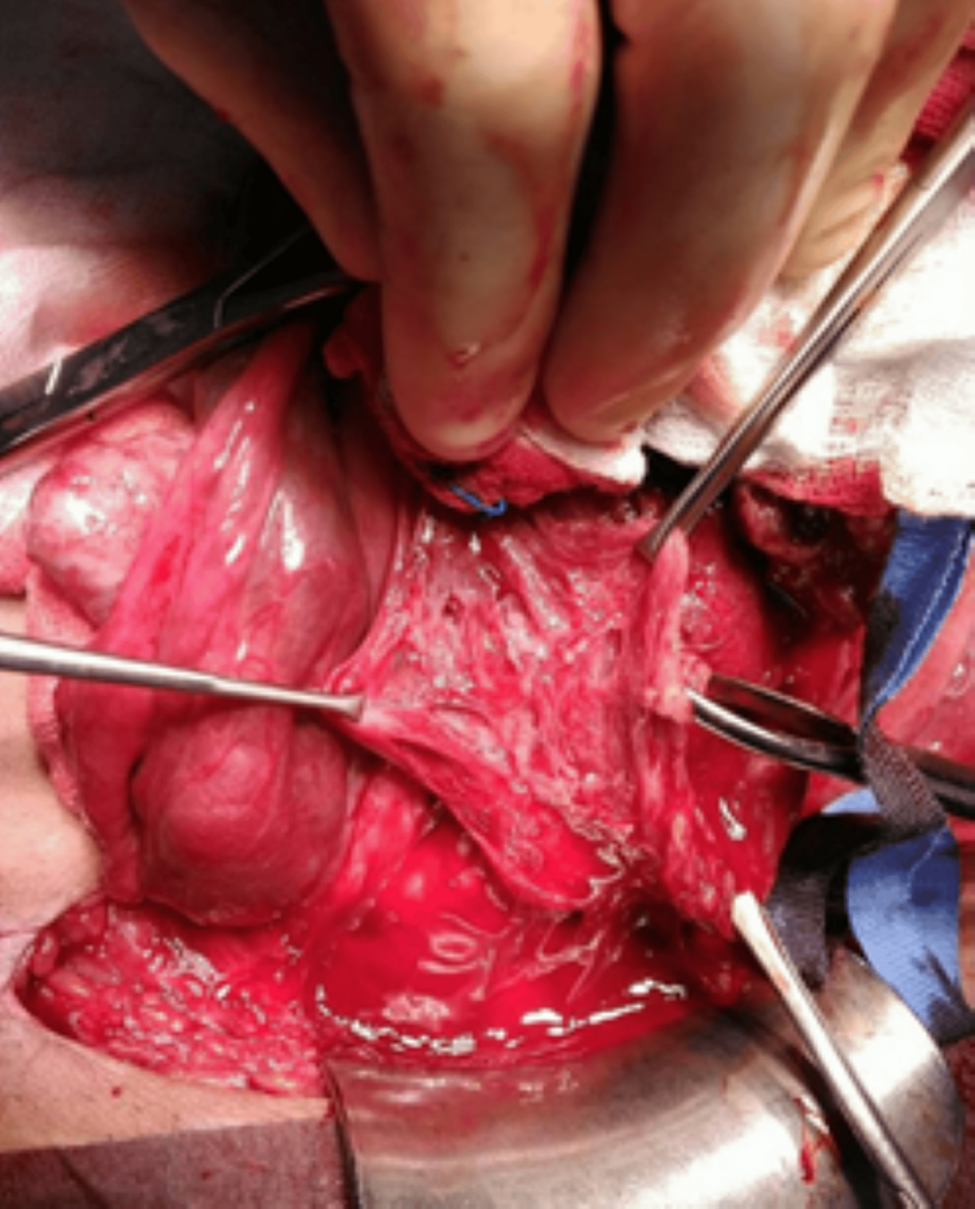
Placental sinuses in the myometrium

The baby was delivered cephalic by making a transverse incision on the uterus through the placenta. The uterus was then exteriorized and the placenta was delivered manually. Bilateral uterine artery ligation was performed and the fenestrated area on the uterine wall showing placental sinuses in the uterine myometrium was partially excised and sent for histopathology (see Figures 4, 5).

**Figure 6:**
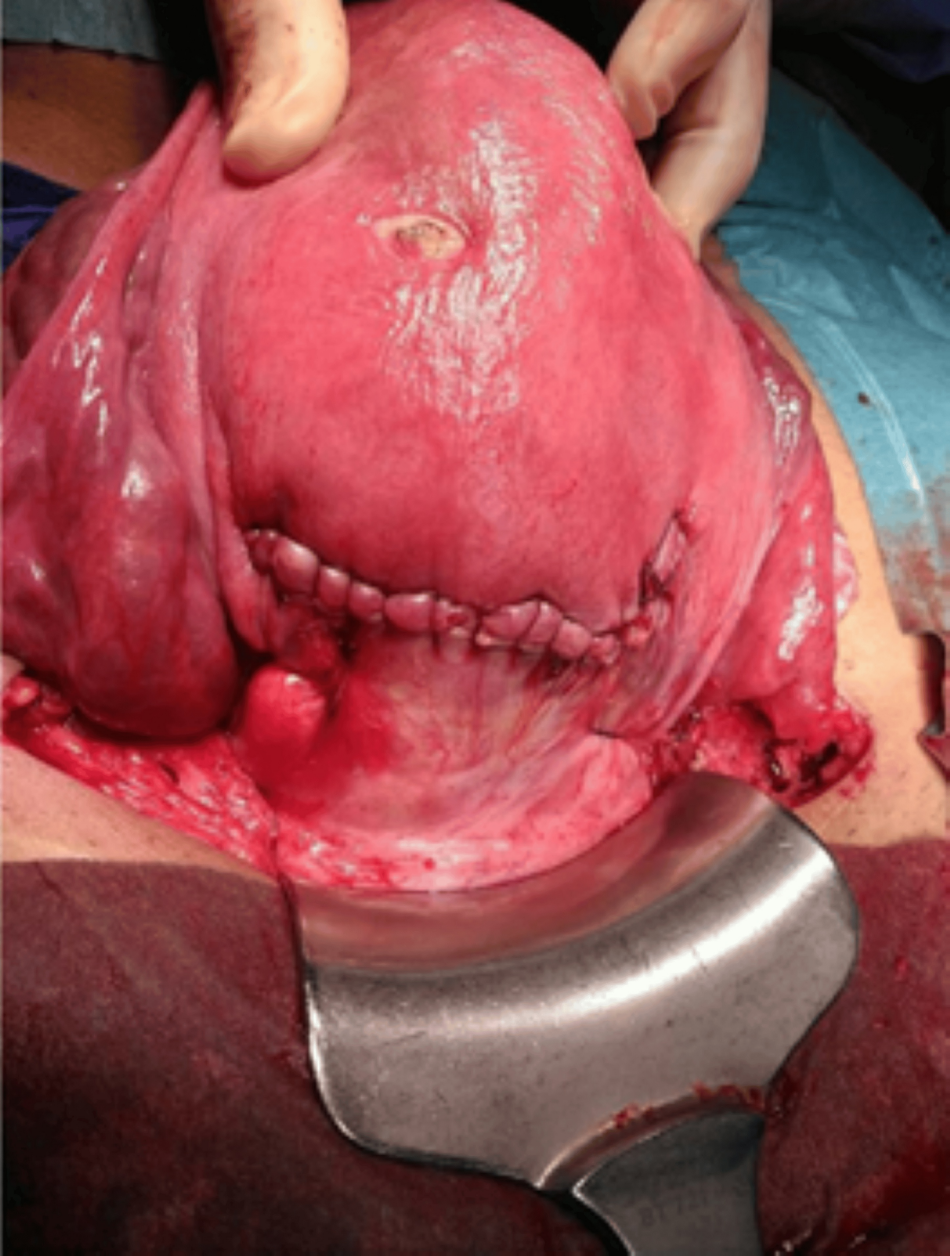
Reconstructed uterine wall

The uterine wall was reconstructed and layers were closed in three layers (see Figure 6). The abdominal wall was closed in layers.

The patient received two units of blood intraoperatively and one unit postoperatively. She was transferred to the ICU for observation in view of Factor 12 deficiency. She was later discharged on day 3 postoperatively.

Histopathology of the excised uterine wall confirmed the intraoperative findings; the report described pieces of myometrial tissue, showing fascicles of smooth muscle bundles with intervening fibro collagenous stromal tissue showing foci of small hemorrhage and variably dilated and congested blood vessels.

## Discussion

PAS represents a range of placental pathologies, including placenta accreta, increta, and percreta, which are differentiated by the extent of placental invasion into the uterine wall. Placenta acreta indicates villi attachment to the myometrium, whereas placenta increta involves villi invasion into the myometrium, and placenta percreta occurs when villi penetrate through the myometrium and into or through the serosa [1]. The condition can lead to severe maternal morbidity due to difficulties in placental separation during delivery, often resulting in massive hemorrhage and the need for a hysterectomy. The pathophysiology of PAS is largely attributed to abnormal trophoblast invasion, which typically occurs at sites of defective decidualization, a process often caused by previous uterine surgery, such as cesarean sections [3,4].

In the case presented, the patient’s history of two prior cesarean sections, combined with a diagnosis of Grade IV placenta previa, placed her at a significantly elevated risk for PAS. Studies have consistently shown that previous cesarean sections are one of the most important risk factors for PAS. The underlying mechanism involves scarring of the uterine wall, which leads to defective decidualization and facilitates abnormal trophoblastic invasion into the myometrium [4,6]. This disrupted interface increases the likelihood of deep placental attachment, resulting in PAS.

In women with placenta previa, the risk of PAS escalates with increasing numbers of prior cesarean deliveries, reaching 3% after one cesarean delivery and escalating to 11%, 40%, 61%, and 67% after two, three, four, and five or more cesarean deliveries, respectively [1].

Clinical features and diagnosis

The clinical presentation of PAS can vary, but common findings include APH and a history of previous cesarean deliveries. The patient in this case presented with APH at 34+4 weeks gestation, which is a classic complication of placenta previa. APH is often seen in PAS cases due to the abnormal placental attachment disrupting the normal uterine contractility and causing premature separation or bleeding [7]. The diagnosis of PAS is confirmed through a combination of clinical risk factors and advanced imaging techniques such as US and magnetic resonance imaging (MRI) (gold standard). US findings, such as loss of retroplacental hypoechogenicity and increased vascularity, are suggestive of PAS [4,7].

MRI is considered an alternative or adjunct to US for prenatal PAS diagnosis, characterized by features such as intraplacental dark bands, abnormal placental or uterine bulge, disruption of retroplacental dark zone, heterogeneous placenta, and abnormal placental vessels. Although MRI’s diagnostic accuracy relative to US remains uncertain, it may prove beneficial in cases with inconclusive US findings, particularly those involving posterior placentae, and in determining the depth of placental invasion [2].

In this patient, the US revealed an anterior placenta with a well-maintained retroplacental hypoechogenic line, which is not a key indicator of PAS. The absence of this line represents the loss of the normal serosal-myometrial interface, a critical feature in diagnosing PAS [4]. The absence of bridging vessels on US was a reassuring sign, as it suggested that the placental invasion had not yet extended through the serosa or beyond the myometrium. This highlights the importance of distinguishing between different degrees of placental invasion, which influences the management plan.

Management and surgical approach

The management of PAS is complex and requires a multidisciplinary approach to minimize maternal and fetal risks. The timing and mode of delivery, as well as the choice of surgical interventions, depend on the degree of placental invasion and the associated risks, such as hemorrhage or preterm birth. For patients diagnosed with PAS, planned cesarean delivery is often recommended, typically around 34-36 weeks of gestation, to minimize the risks of spontaneous labor and hemorrhage [5]. In this case, the patient was prepared for an elective cesarean section after being counseled on the associated risks, including massive blood loss and the potential need for hysterectomy.

During the surgery, a transverse incision was made on the uterus through the placenta, allowing for the delivery of the baby. The decision to perform bilateral uterine artery ligation prior to placenta removal was made to reduce the risk of hemorrhage, which is a common complication of PAS. The placental mass was manually removed, and the area of focal adherence to the uterine wall was sent for histopathological examination. This approach reflects current best practices for managing PAS, as uterine artery ligation has been shown to be an effective method of controlling bleeding without the need for hysterectomy in certain cases [7].

Postoperative care

Postoperative management in PAS cases requires careful monitoring for bleeding, particularly given the patient's Factor 12 deficiency, which complicates hemostasis. Factor 12 deficiency is a rare clotting disorder that increases the risk of bleeding complications. The patient received two units of blood intraoperatively and one unit postoperatively. She lost an estimated 1000 mL of blood and was transferred to the ICU for observation, a necessary precaution in such cases to ensure prompt management in the event of further hemorrhage. Her postoperative recovery was uneventful, and she was discharged on day 3 postoperatively, which is consistent with the typical recovery time for patients who undergo complex surgeries like cesarean delivery for PAS.

Histopathology

The histopathology findings from the excised tissue confirmed the diagnosis of PAS, with the specimen showing myometrial tissue with areas of hemorrhage, vascular congestion, and dilated blood vessels, consistent with abnormal placental adherence and invasion. Importantly, no malignancy was identified, ruling out the possibility of trophoblastic neoplasia or other malignant processes. The presence of hemorrhagic areas and abnormal blood vessels is characteristic of PAS and underscores the importance of histopathological examination in confirming the diagnosis and ruling out other conditions [8,9].

## Conclusions

In conclusion, this case underscores the critical importance of early diagnosis, careful surgical planning, and multidisciplinary management in addressing PAS, particularly in patients with risk factors such as previous cesarean deliveries and placenta previa. The patient’s history of multiple cesarean sections placed her at a significantly elevated risk for PAS, a condition that can result in severe maternal morbidity, including massive hemorrhage. Advanced imaging, such as ultrasound, was essential for diagnosing PAS and determining the degree of placental invasion, which guided the surgical approach. The successful management involved planned cesarean delivery with uterine artery ligation to control bleeding, alongside vigilant postoperative care. Histopathological examination confirmed the diagnosis and excluded malignancy, further emphasizing the importance of a comprehensive, individualized approach to optimize outcomes. This case highlights the need for timely intervention and thorough monitoring to mitigate the potential complications of PAS and ensure the best possible maternal and fetal outcomes.

Recent data indicate a rising prevalence of PAS disorders, underscoring the critical need for expanded research to enhance early diagnosis, refine critical management, and reduce associated maternal morbidity and mortality.
